# Supplementary material for: Multiplex nodal modularity: A novel network metric for the regional analysis of amnestic mild cognitive impairment during a working memory binding task
Source: PLoS One. 2025 Aug 22;20(8):e0328736. doi: 10.1371/journal.pone.0328736 (PMC12373287; doi:10.1371/journal.pone.0328736)
Supplement: S1 Appendix — (PDF) [file pone.0328736.s001.pdf]

fMRI windowing

During fMRI scanning, stimuli onset does not always line up perfectly with volume acquisition (i.e., a stimuli could be shown at 1.5s while the volume was acquired from 0-2s). We account for this by approximately aligning our encmaint and probe windows to stimuli onset and optimize to capture peak HRF. First, we compare onset of stimulus timings with the following trial and shift our window forward or backward a volume to minimize the offset of trial start and volume acquisition time. This resulted in an offset for our task phase window in the range $[-1.47\text{s}, 0.24\text{s}]$. We then shift forward our window by one volume (2s), resulting in an offset of $[0.53\text{s}, 2.24\text{s}]$. The reason to shift forward is to better capture the signal peak of the HRF. To illustrate this shift's interaction with the HRF, consider the 4s probe phase while accounting for the 2s shift to increase the separation between the two phases. This results in our images corresponding with the signal dynamics of the task from stimuli onset up until a point in the range $[6.53\text{s}, 8.24\text{s}]$. This ensures that the signal peak information occurring at 5s is captured. It is important to note that there are a very small number of samples that do not perfectly capture the HRF peak. This occurs when the maintenance phase is 2s and the alignment of the task phase window is towards the lower bound of our offset in the range $t = [4.53\text{s}, 6.24\text{s}]$. Given that the number of cases where our window does not fully capture the HRF peak is very small, and that the offset is only 0.47s, we maintained these samples, acknowledging that in a small number of cases the analysed sample does not fall exactly on the peak of the HRF. This would very slightly reduce our ability to find differences due to stimuli in the encmaint phase of the task but we expect this effect to be minor.

Variable maintenance phase of the VSTMBT

The maintenance phase, where subjects must remember the presented shapes or coloured shapes, is displayed for a variable time as discussed in Fig 1. This is part of the fMRI design optimization aimed at decoupling the BOLD signal from encoding and maintenance for analyses such as statistical parametric mapping (not explored in this study). In this study, we do not expect the variable maintenance window to appreciably influence the comparison of nodal modularity in healthy and diseased brain networks due to the following. First, each subject's encmaint brain network is constructed from the correlations between repetitions of combined instances of the encoding and maintenance phase preserving temporal variability between brain ROIs. Second, all subjects in the study had an equal number of trials of each maintenance phase length presented at random within the scanning session. This ensures that the total time of all maintenance phases for each subject is equal.
