# Supplementary material for: Multiplex nodal modularity: A novel network metric for the regional analysis of amnestic mild cognitive impairment during a working memory binding task
Source: PLoS One. 2025 Aug 22;20(8):e0328736. doi: 10.1371/journal.pone.0328736 (PMC12373287; doi:10.1371/journal.pone.0328736)
Supplement: S2 Appendix — (PDF) [file pone.0328736.s002.pdf]

Description of publicly available datasets

Zachary's Karate Club is a commonly used dataset for exploring community structure in networks [1, 2]. It is a social network modelling the split in a karate club with one leader leaving and forming their own club and taking half of the members with them. In this case, the network's nodes are people and the edges between them denote friendship. Data was accessed (16/02/2024) and downloaded from `http://konect.cc/networks/ucidata-zachary/`.

The NKI-Rockland cohort is a publicly available dataset containing 196 subjects across lifetime (114 male; age range: 4-89 y.o.) [3] of pre-processed rs-fMRI and DTI data. These were generated at 3T with the following: an acquisition time of 10:55, TR = 2500ms, TE = 30ms, voxel size = 3mm^3, and on 38 slices. DTI had an acquisition time of 13:32, TR = 10000ms, TE = 91ms, voxel size = 2mm, and on 58 slices. Each of the connectomes were parcellated into 188 regions of interest using the Craddock atlas. From these, the network edge weights were computed using Pearson correlation and were normalized by the maximum edge weight to the range [0,1]. In the case of DTI, edge weight was determined by the number of fibers that intersected at least one voxel in both the source and target region of interest (ROI) and normalized with the same method as with the fMRI networks. For further information, pre-processing, and availability see [4] (accessed on 30/08/2021).

Using the provided rs-fMRI and DTI matrices above, we process each subject's data by taking the absolute values of the fMRI connectivity networks and threshold them. Edges with the lowest edge weight were removed until the fMRI network's density matched that of the the subject's corresponding DTI network. Matching densities in this way helps to minimize some modality specific weighting of modularity within a multiplex setting, as fMRI networks are inherently much denser than DTI networks. However, as will be seen in the results, the two modalities retain very different topologies as expected even after matching for density.

References

1. Zachary WW. An Information Flow Model for Conflict and Fission in Small Groups. Journal of Anthropological Research. 1977;33(4):452–473. doi:10.1086/JAR.33.4.3629752.

2. Girvan M, Newman MEJ. Community structure in social and biological networks. Proceedings of the National Academy of Sciences. 2002;99(12):7821–7826. doi:10.1073/PNAS.122653799.

3. Nooner KB, Colcombe SJ, Tobe RH, Mennes M, Benedict MM, Moreno AL, et al. The NKI-Rockland sample: A model for accelerating the pace of discovery science in psychiatry. Frontiers in Neuroscience. 2012;0(OCT):152. doi:10.3389/fnins.2012.00152.

4. Brown JA, Rudie JD, Bandrowski A, Van Horn JD, Bookheimer SY. The UCLA multimodal connectivity database: a web-based platform for brain connectivity matrix sharing and analysis. Frontiers in Neuroinformatics. 2012;0(NOV):28. doi:10.3389/FNINF.2012.00028.
