# Supplementary figures and images for: Multiplex nodal modularity: A novel network metric for the regional analysis of amnestic mild cognitive impairment during a working memory binding task
Source: PLoS One. 2025 Aug 22;20(8):e0328736. doi: 10.1371/journal.pone.0328736 (PMC12373287; doi:10.1371/journal.pone.0328736)

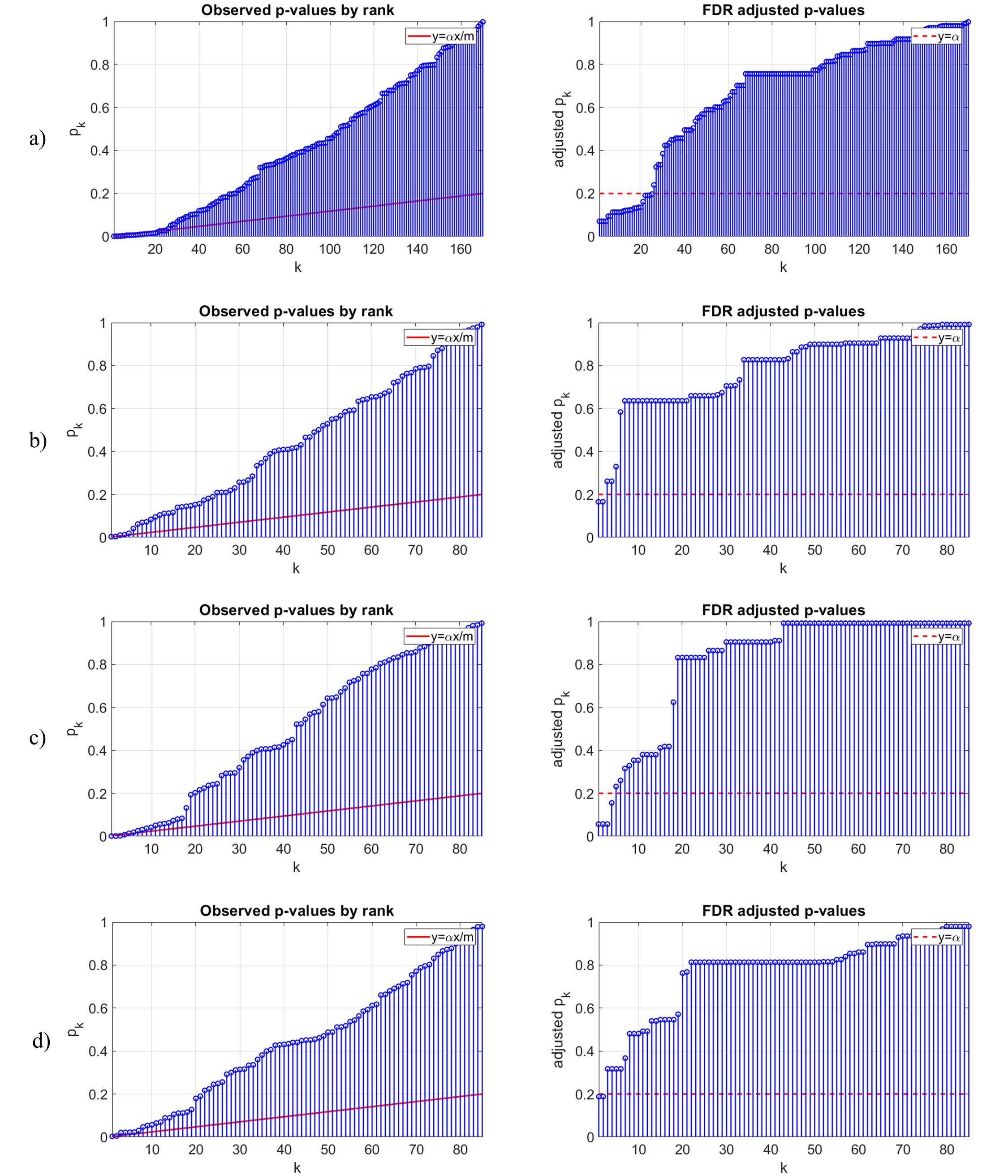

Supplement: S1 Fig — This figure visualizes the Benjamini-Hochberg FDR correction for each of our comparisons. Plots of observed p-values (pk) by rank (k) show the ranked p-values in our ROI comparisons of a) fMRI multiplex control vs. MCI converters, b) DTI control vs. MCI, c) DTI control vs. MCI converters, and d) DTI eMCI vs. MCI. FDR controlled p-values are all pi from i = 1...k where pk≤αkm and α is our chosen threshold. Plots of FDR adjusted p-values are also given to more easily visualize those that pass FDR correction (those that fall under the dashed line at α=0.2). (TIF) [file pone.0328736.s003.tif]

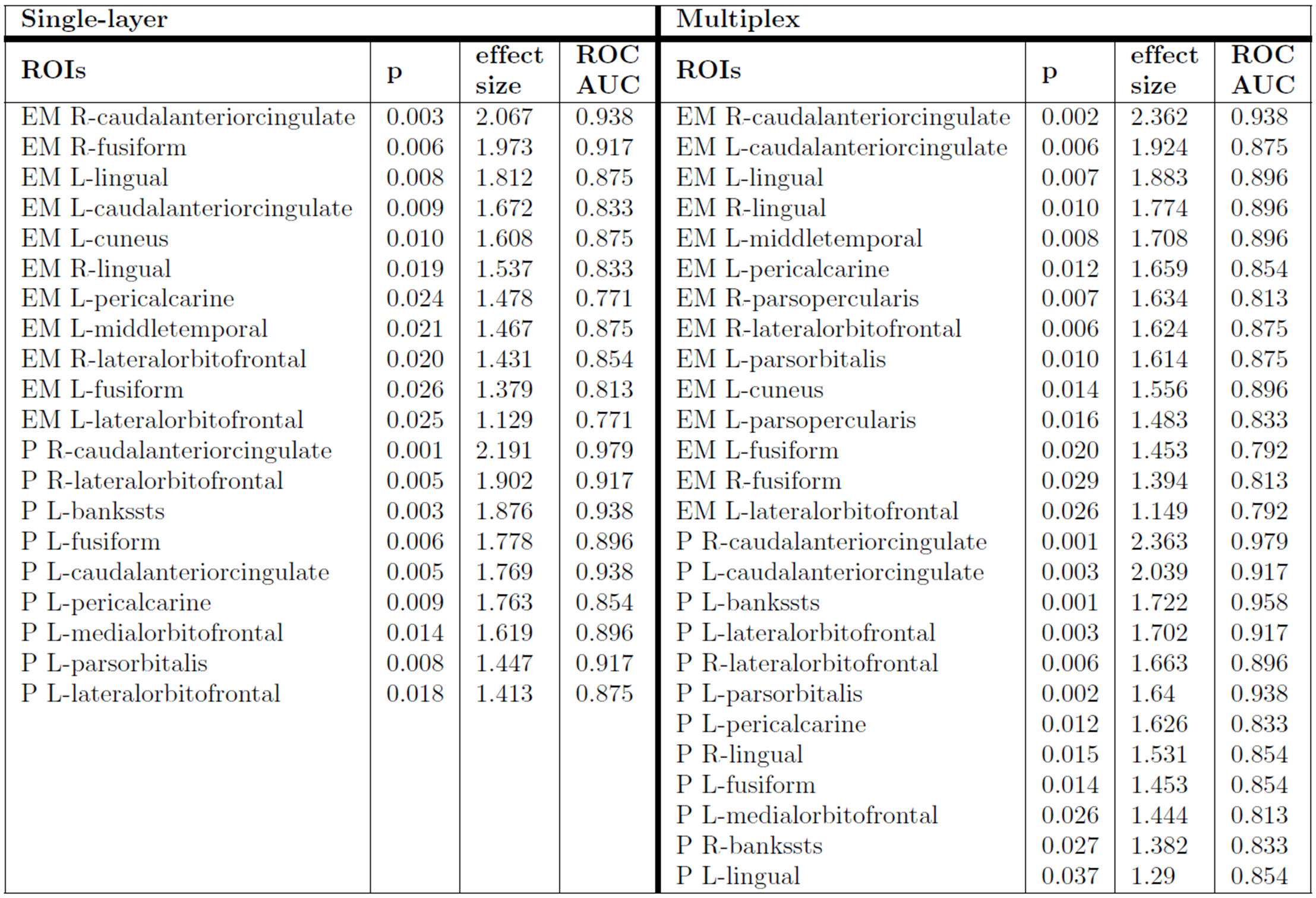

Supplement: S1 Table — This table displays the ROIs which pass the thresholds of p≤0.05 and where p-values are controlled by FDR at α=0.2. These ROIs reside in either the encmaint (EM) or probe (P) single-layers or modeled together as a multiplex network (results given seperately in the left and right sides of the table respectively). Standard p-value (p) and effect size is displayed following permutation test and the area under the curve (AUC) of the Receiver Operating Characteristic (ROC). Multiplex construction yields a higher number of ROIs which pass the statistical thresholds (25 vs. 20), with lower p-values and higher effect sizes and ROC AUC among most ROIs. Additionally, note that FDR is applied to each layer separately in the single-layer case (85 ROIs each). Consequently, and since FDR is more strict for a higher number of comparisons, the multiplex model displays higher statistical power in determining regional changes in nQ between controls and MCI converters. (TIF) [file pone.0328736.s004.tif]

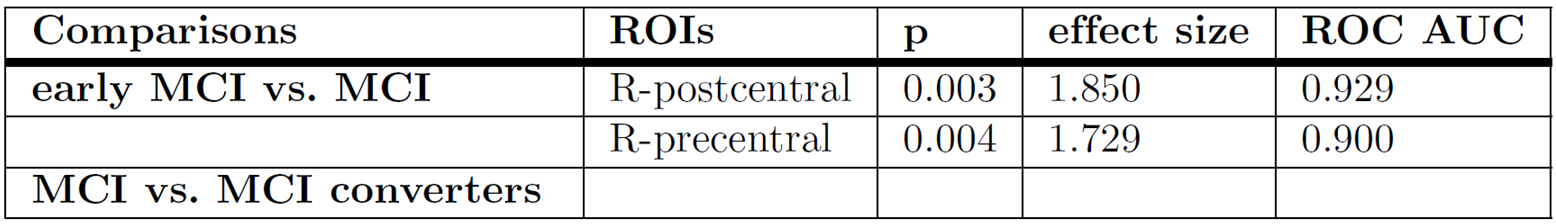

Supplement: S2 Table — This table displays the ROIs which passed p≤0.05 and FDR controlled at α=0.2. L and R indicate the left or right hemispheres of the brain respectively. Standard p-value and effect size is displayed following permutation test and the area under the curve (AUC) of the Receiver Operating Characteristic (ROC). (TIF) [file pone.0328736.s005.tif]

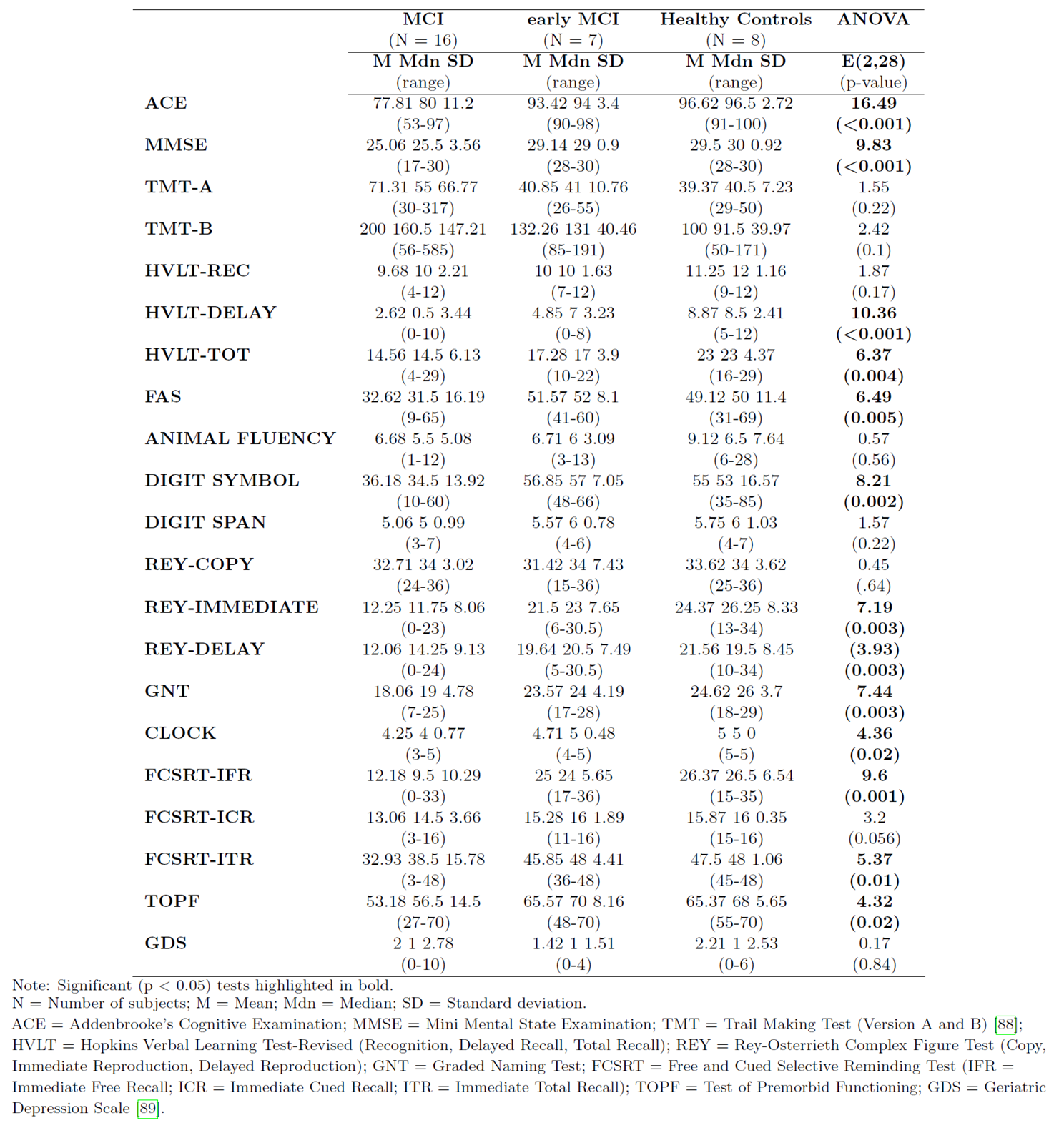

Supplement: S3 Table — shows the neuropsychological profile of MCI patients, early MCI patients and healthy controls entering the study. ANOVA revealed that patients with MCI performed poorer than healthy controls on ACE [76], MMSE [76], HVLT-DELAY and TOT [77], FAS [78], DIGIT SYMBOL [79], REY-IMMEDIATE and DELAY [80], GNT [81], CLOCK [76], FCSRT-IFR and ITR [82], and TOPF [83]. More specifically, significant differences emerged from comparisons between MCI patients and healthy controls, and MCI patients versus early MCI patients overall. HVLT was carried out poorly from both MCI and early MCI patients compared to the control group, whereas Rey figure delayed copy was significantly underperformed by MCI patients only. Although the conversion to AD in some patients has been ascertained once the collection of neuropsychological data was done, and MCI converters have not been taken into account here, we can conclude that these results are in line with clinical diagnosis and reflect the progression of the disease through the spectrum. (TIF) [file pone.0328736.s006.tif]
