# Supplementary material for: Multiplex nodal modularity: A novel network metric for the regional analysis of amnestic mild cognitive impairment during a working memory binding task
Source: PLoS One. 2025 Aug 22;20(8):e0328736. doi: 10.1371/journal.pone.0328736 (PMC12373287; doi:10.1371/journal.pone.0328736)
Supplement: Attachment — Submitted filename: Response to Reviewers.pdf [file pone.0328736.s007.pdf]

Overview of changes:

We thank the Reviewers and the PLOS One editorial team for their support and suggested changes. Here we will outline the substantial changes to the manuscript following the Reviewer suggestions:

- The results and discussions sections have been heavily restructured.
- Results have significantly changed following FDR correction - leading to the reduction of the number of figures and breadth of discussion. We hope that these changes promote a clearer, more robust, and concise demonstration of our results.
- Several sections have been substantially condensed (introduction and method sections), reducing unnecessary details in the main text, and with additional changes in sub sectioning for clarity.
- Finally, we aimed to address concerns with our communication around the limitations in sample size which we hope are now made more clear within the text, and made less worrisome with improved statistical stringency with FDR correction given that results remain strong and the main conclusions of the study do not change.

Thank you once again for your support. We believe that your comments have resulted in a stronger manuscript that will be of interest to the readership of Plos One.

Please note that tracked changes were done automatically in LaTeX. As such, some tables in the marked version of the new manuscript have poor formatting. However, note that they are properly formatted in the clean version.

Reviewer #1:

Reviewer's Comment

In this paper, Authors propose a new metric for assessing community structure in various single-and multi-layer networks, called nodal modularity (nQ). Contrary to the more traditionally used modularity, nQ not only quantifies community structure at a global level in a network, but also provides some information about community structure at a meso-scale level (i.e. at level of single regions). NQ was tested for calculation using functional MRI (fMRI) and diffusion tensor MRI (DT MRI) data from a group of mild cognitive impairment (MCI) patients and healthy controls, calculating multiplex nQ across functional/structural layers. Results suggest that observed changes in nQ in MCI align with expected trajectories of changes in alzheirmer's disease, suggesting applicability of nQ as a biomarker for the disease.

The paper is of interest, but it overall very long, with several redundant and unnecessary information being reported; several sections can be considerably condensed. Following major issues should be addressed.

Author's response:

We thank you for your feedback and agree with the above assessment of the manuscript. We have aimed to address the points below with the length of the manuscript in mind. Specifically, in condensing the introduction, methods, results, and discussion sections (the final two as a result of higher statistical stringency). We hope that the new manuscript is a more acceptable length.

Reviewer's Comment

1) The Abstract repeatedly states that nQ is very useful (compared to standard modularity) because it is a more fine-grained metric giving information not only at global level but a mesoscale level. Despite this, the results reported in the Abstract are very generic and do not describe which are the regions showing abnormal nQ. Since Authors claim that this is one of the major novelty of their study, I think that results should be expanded in this direction.

Response: We agree with the Reviewer about the need to include more detailed results in the Abstract. After restructuring the results and discussion sections, including improved statistical stringency based on Reviewer 2's feedback, we have included more specific

wording in the abstract regarding individual ROIs and their changes in nQ. Due to the high number of ROIs that were detected as statistically significant, and that many of them reside in common subsystems where deposits of amyloid-beta and tau are observed, we choose to report results in the abstract at the level of subsystems due to word count constraints. We have also shortened the abstract in several places to accommodate these changes.

Reviewer's Comment

2) The first section of the introduction is excessively long and includes a lot of information about Alzheimer's pathology (unrelated to neuroimaging) that is mostly background and not necessary for the paper. This section should be shortened. In general, the methods section is also overly detailed and can be significantly condensed. The same applies to the number of figures and tables.

Response: We thank the Reviewer for the detailed suggestions to make the article more concise. We agree, and have removed the paragraph on Alzheimer's pathology (2nd paragraph in the Introduction of the previous version of the manuscript). In the methods section, we have removed the description of classical modularity as it is well known, condensed the section on nodal modularity for clarity, and moved important points on modularity maximization to a separate subsection "Modularity maximization". This makes the section shorter and better structured. Additionally, the number of figures and tables across the main text and supplemental material have been reduced following stricter statistical criteria raised by Reviewer 2.

Reviewer's Comment

3) The end of the Introduction is not really introduction, it anticipates already results and conclusions. All the last paragraph of the introduction reporting results and conclusions should be deleted.

Response: The last paragraph of the introduction has been deleted. Thank you.

Reviewer's Comment

4) One of the main limitations of the paper is the very small sample size of HC and MCI groups included. This should be clearly stated in the paper.

Response: We have added a statement on this to the abstract ("While limited by sample size, changes in nQ for individual regions of interest (ROIs) in our fMRI networks were predominantly observed in visual, limbic, and paralimbic systems in the brain, aligning with known AD trajectories and linked to amyloid-β and tau deposition.") and note its presence in the "Application of nQ to investigate local scale changes in MCI " portion of the methodology section on the choices of statistical tests (now improved with FDR, lines

399-422 in the new version of the article) and in the discussion (lines 604-605) and conclusion sections (line 651) of the paper.

Reviewer's Comment

5) It is not clear which version of SPM software was used for fMRI analysis, some paragraphs mention SPM8 and other paragraphs mention SPM12. SPM8 is a very old software, so it is surprising that Authors use it for some analysis. To be sure that Authors are using appropriate software for the analysis, please also report software versions for Freesurfer and FSL.

Response: We are thankful for this comment. We acknowledge the two versions of SPM as an error in referencing. Specifically, in the prior version of the manuscript it states "Using SPM8 (SPM, https://www.fil.ion.ucl.ac.uk/spm/), fMRI pre-processing follows as in [34]." Ref 34 uses SPM8, and while this paper uses the same pre-processing pipeline, the version of SPM was overlooked. The current paper follows ref 34's pre-processing pipeline but with SPM12 and the new version of the manuscript has been updated to reflect this. Furthermore, Freesurfer v5.3.0 and FSL v6.0.1 versions have now been reported on lines 146 and 148 respectively in the new version of the manuscript.

Reviewer's Comment

6) The paragraph in the method about modularity is very long and can be condensed. The same applies for the description of the very standard network metrics (e.g. degree, clustering coefficient, centrality..). these are all very standard metrics which were described in details in hundreds of previous publications.

Response: The modularity section has been removed given that classical modularity has been well studied. Important details have been moved to a separate subsection of the methods "modularity maximization" (line 285 of the revised manuscript). The same has been done on the network metrics, only including detail on the multiplex versions of the measures. See lines 335-345 in the revised manuscript.

Reviewer's Comment

7) I don't understand how the fact that nQ is not correlated with the other graph metrics constitutes a "verification" of nQ. It only tells us that it is independent, but it certainly does not ensure that it is meaningful.

Response: We thank the Reviewer for this observation. We have carefully revised the text to avoid the term "verification" in this context. We now present the results in subsections "Comparison of nQ with other nodal graph measures" (line 330 of the revised manuscript)

and “Independence of nQ with other nodal graph measures “ (line 449 of the revised manuscript).

Reviewer’s Comment

8) Authors should provide a Results section clearly separated from the Discussion section. In the present paper version, the two sections are combined in a very confusing way.

Response: We have restructured the results and discussion into separate sections and have improved sub sectioning to improve clarity. Additionally, note that this section has also undergone substantial changes due to results changing in accordance to FDR correction.

Reviewer’s Comment

9) Please delete the wording “disease progression” from any result and interpretation; “progression” implies a longitudinal assessment over time, while the present study is cross-sectional.

Response: We are thankful for this insight. We have removed mention of “disease progression” when referring to results and interpretation.

We hope that the revised manuscript will address all your concerns fully.

Reviewer #2:

This study introduces nodal modularity (nQ), a novel network metric designed to evaluate particular changes in brain network community architecture, particularly in the context of amnestic mild cognitive impairment (aMCI) and Alzheimer's Disease (AD). The approach employed in this research is both captivating and technically solid, providing significant insights for the realms of neuroimaging and network neuroscience. The authors receive commendation for merging functional and structural data in an innovative analytical model and validating their techniques with established datasets.
However, I would like to highlight several concerns and limitations that I believe require the authors' attention and clarification before this manuscript.

Authors' response:

We thank the Reviewer for highlighting their concerns and the limitations of this study. Specifically, we have implemented the suggested FDR correction and hope that this provides sufficient statistical stringency given that results remain strong. We also hope that it is now more clear within the text the sample size limitations of this paper, along with other changes aimed at improving the clarity of explanations and impact. We hope that the new manuscript provides a clearer and more robust study in the face of its limitations.

Major Concerns

Reviewer's Comment

1. Limited Sample Size and Power
The number of subjects across groups — particularly in the early MCI and MCI converter subgroups — is relatively small. This naturally limits the statistical power and raises concerns about the robustness of the results. While the authors apply permutation testing and ROC analysis, the reliability of node-level inferences across so many comparisons is still questionable in the absence of adequate correction for multiple comparisons.

Response: In accordance with a comment by Reviewer 1, we have improved clarity by mentioning the limited sample size in the Abstract and note its presence across methodology, discussion, and conclusion sections. In regard to multiple comparisons, we will address this in detail in point 2. In brief, we have applied Benjamini-Hochberg FDR correction to the node-level statistical tests across all of our results and have updated the new manuscript's results and discussions to reflect this.

Reviewer's Comment
2. Absence of Multiple Comparison Correction
Given the large number of regions (85 ROIs) evaluated independently for significance, the study is at risk for inflated false positives. The authors have not clearly stated whether false discovery rate (FDR) correction or any other multiple testing correction was applied. For a study aiming to identify diagnostic biomarkers, statistical stringency is crucial.

Response: Initially, we had used ROC AUC given that [1] argues that discriminative power is more linked to effect size and variability than group size. This was especially important given that our low sample size and early stage of AD limits power which in turn limits the effectiveness of applying FDR (given that the strictness of control is driven by the number of comparisons which is 170 ROIs in the multiplex model). However, we acknowledge the need for multiple comparison correction and have applied Benjamini-Hochberg FDR at $\alpha = 0.2$ to all results, updating the figures, tables, and discussions to reflect this. We additionally note that this is a pilot study demonstrating nQ as a novel measure of nodal community structure and given the limited sample size we cannot claim diagnostic capability. We hope that the updated discussion section reflects this more clearly.

Of note, a requirement for the popular Benjamini-Hochberg FDR to properly control false positives is independent or positively dependent comparisons. Given that nQ is a nodal measure of group structure (within module comparisons are expected to be positively correlated) and Q increases across the stages of AD, we expect this requirement to be met in the majority of cases. However, due to the complexity of biological networks and AD's effect on brain network reorganization, we cannot rule out the possibility of some negative dependencies between p-values and acknowledge this as a limitation. The authors explored other options of multiple comparison correction that were appropriate for arbitrary dependency structures, but found that many are known to be too conservative in the literature [2], or were not appropriate for testing nodal hypotheses (such as FWER) [3]. In lieu of no better alternative, and the need to control for false discoveries for a large number of comparisons, we elect to use Benjamini-Hochberg FDR and note its prominent use in the literature even for cases where arbitrary dependency structures may be present.

Additionally, see lines 399-422 of the new manuscript for the description of the usage of FDR.

[1] https://doi.org/10.1002/epd2.20010

[2] https://www.jstor.org/stable/2674075

[3] https://doi.org/10.1016/j.metip.2023.100120

Reviewer's Comment

3. Biological Interpretability of nQ
While nQ is mathematically well-defined, its biological interpretation remains somewhat abstract. It is not clear how variations in nQ values across brain regions translate into functional implications or pathophysiological mechanisms. A clearer, neurobiologically grounded discussion of what an increase or decrease in nQ implies would significantly enhance the manuscript's impact.

Response: We have added clarity on the biological interpretation of nQ in brain networks in the following part of the discussion section (lines 549-564 in the revised manuscript).

Reviewer's Comment

4. Generalizability of Findings
The specificity of the Visual Short-Term Memory Binding Task (VSTMBT) is well established for early AD detection; however, the utility of nQ outside this specific paradigm remains unexplored. The manuscript would benefit from a discussion of how transferable this method might be across tasks, domains, or even other clinical populations.

Response: We are grateful for this suggestion. We have added discussion of nQ's applicability to other biological networks and beyond in other domains. While a more extensive discussion would be beneficial, the authors acknowledge the other reviewers' concerns on the length of the manuscript and keep it as a brief overview of potential applications.

Reviewer's Comment

5. Data Availability and Reproducibility
A portion of the neuroimaging data used in this study is not publicly available due to clinical restrictions. While this is understandable, it poses a challenge to reproducibility — particularly in a journal like PLOS ONE, which emphasizes open science. It would be helpful if the authors could at least share simulated data or a synthetic version of the dataset for validation of their pipeline.

Response: Unfortunately, the study is on very strict data confidentiality restrictions and there are legal implications to sharing the raw data, including extracts made from this data. However, note that access to some of the contents of the secondary data (current results) may be available on individual request from Javier Escudero (javier.escudero@ed.ac.uk) or Mario A. Parra (mario.parra-rodriguez@strath.ac.uk), and that clinical research access to

raw data can be applied for through the NHS Lothian Health Board. We have updated the S1 Appendix to clarify this (lines 679-686 in the new manuscript).

There are legal restrictions in sharing the de-identified data set. These were imposed by the Research & Development department of the NHS Lothian Health Board - Tel: (+44) 0131 242 3330). Specifically, this data is held under their “Confidentiality and Disclosure of Information” policy as follows:
You must not divulge Confidential Information to any third party during the period of your research or any time thereafter without the proper authority having first been given. All Confidential Information belonging to the Board, together with any copies or extracts thereof, made or acquired by you in the course of research shall be the property of the Board and must be returned to the Principal Investigator on completion of the research to which they relate or on the termination of your employment whichever is the earlier date. You will be entitled to retain any copies or extracts made or acquired by you in the course of research for references purposes only, provided that such copies or extracts are held and maintained in accordance with the provisions of the Data Protection Act 2018 and Caldicott principles.
Thus, data cannot be shared publicly without clinical research access approval from NHS Lothian and cannot be shared with any 3rd party, including extracts made from the data, given that the raw data is part of an ongoing study under the Principal Investigator Mario A. Parra.

Reviewer’s Comment
Minor Comments
• The description of nQ is technically dense. Including an more in-depth illustrative figure or schematic could help readers better visualize the interplay of layers, modules, and nodal contributions.

Response: Given length concerns of the paper, specifically in regard to the number of figures posed by the other reviewer we elect not to add another one. However, we point the reviewer to Fig 2. which has been updated and the discussion contained within. We have revised this part of the manuscript to better reflect its connection to nQ and hope that the additional example better clarifies the interplay between connectivity, modules, and how changes in these across layers affect nodal contributions to modularity.

Reviewer’s Comment
• The discussion of the correlation between nQ and other network metrics (e.g., degree, PageRank, clustering coefficient) is informative; however, the manuscript could further elaborate on the implications of these relationships

Response: The goal of these tests were to determine that nQ was independent from other common measures of node importance. Given that the manuscript is already lengthy, and that the focus of the study is on AD, the authors have elaborated further but kept it brief.

Reviewer's Comment
Recommendation
Addressing the statistical concerns, clarifying the biological interpretation of the metric, and improving transparency around data availability will enhance the clarity and impact of this study.

Response:

Thank you for your assistance in improving the manuscript. While limited by sample size along with data availability, we hope that the improved statistical stringency and clarity surrounding nQ have created a more robust and compelling paper. We hope that the revised manuscript has addressed your concerns and thank you once again for your support.
