# Supplementary material for: Multiplex nodal modularity: A novel network metric for the regional analysis of amnestic mild cognitive impairment during a working memory binding task
Source: PLoS One. 2025 Aug 22;20(8):e0328736. doi: 10.1371/journal.pone.0328736 (PMC12373287; doi:10.1371/journal.pone.0328736)
Supplement: Attachment — Submitted filename: Response to Reviewers #2.pdf [file pone.0328736.s008.pdf]

Reviewer #2:
Upon evaluating the revised submission alongside the authors' detailed point-by-point responses, I am pleased to acknowledge that the majority of my major concerns have been addressed in a satisfactory and transparent manner.

Authors' response:
We are grateful for your feedback and pleased to read that the manuscript has improved substantially in the revision and that the majority of your concerns have been satisfied along with all concerns of Reviewer #1. We hope that our introduction of nQ as a novel measure of nodal community structure brings a notable contribution to PLOS One.

Generalizability and Broader Application
The authors have briefly addressed the applicability of the nQ metric to other paradigms and clinical populations. While the discussion could be more detailed, the current additions are appropriate given the manuscript's length and scope.

Authors' response:
Thank you. We are pleased that this has been addressed given the scope of the paper.

Correction for Multiple Comparisons
While the authors have correctly implemented the Benjamini–Hochberg false discovery rate (FDR) correction across all node-level statistical tests, the choice of an α threshold of 0.2 raises concerns. This threshold is notably more lenient than the conventional levels (e.g., 0.05 or 0.1) and may substantially increase the risk of false positives, potentially compromising the reliability of the findings. Although the exploratory nature and high dimensionality of the analysis are acknowledged, a threshold of 0.2 should be more thoroughly justified, including discussion of the implications for the interpretation of results. Alternatively, the authors might consider adopting a more stringent FDR threshold to balance sensitivity and specificity more effectively. Without such justification or adjustment, the statistical rigor and robustness of the conclusions remain questionable.

Author's response:

We appreciate the Reviewer's feedback. While 0.2 is a moderate threshold for FDR correction, this value has also been used in other imaging studies such as [1,2,3], and specifically in the context of pilot or more exploratory studies [2]. Moreover, we would like to express concern over the potential for false negatives in lowering the threshold any further. To illustrate this point, in the right panel of S1Fig(a), 25 ROIs pass FDR at 0.2, meaning that up to 20% (5 out of 25) of these ROIs may be false positives. If we were to lower the threshold to 0.1, only 3 ROIs would pass FDR correction (of which 10% of those could be false positives). This strongly suggests that 0.1 is too strict a threshold for the data given

the large drop off in results, which are not proportional to the change in threshold, and thus could be inducing false negatives within the results. This is a concern especially for pilot studies such as this where limited power is at high risk for false negatives in multiple comparison correction [4]. We have already acknowledged the limited sample size of the study, which in turn means that discriminative power is driven more by effect size than p-value. Of note, FDR correction does not consider effect size or sample sizes [5].

In the previous version of the manuscript, we had stated on lines 502-504 “Note that since we are applying FDR at $\alpha = 0.2$ that up to 20% of our ROIs may be false positives (equating to 5 ROIs in the multiplex model and 4 ROIs in the single-layer case)”. However, we acknowledge that this was previously limited to the results section only and not explored in the discussion. We have added the following on lines 577-578 “... (note that we focus on general changes given that up to 5 ROIs may be false positives attributed to FDR correction at $\alpha = 0.2$)” in our neuroanatomical exploration of fMRI section (of the revised manuscript).

Thank you for your review and we hope that this justification and change to the manuscript addresses your concerns.

[1] 10.3389/fnhum.2013.00467
[2] https://doi.org/10.1371/journal.pone.0093623
[3] https://doi.org/10.1038/s41598-024-67177-5
[4] https://doi.org/10.1016/j.metip.2023.100120
[5] https://doi.org/10.1002/epd2.20010
